# Children’s preferences among six novel moxifloxacin and linezolid-dispersible tablet formulations

**DOI:** 10.5588/ijtldopen.24.0546

**Published:** 2025-04-09

**Authors:** G. Hoddinott, H.R. Draper, N. Vanqa, S. Myeni, S. Staples, T. Sachs, M. Raffique, N. Tshethu, M. Palmer, L. Viljoen, K. Inabathina, R. Taneja, A.C. Hesseling, A.J. Garcia-Prats

**Affiliations:** ^1^ Desmond Tutu TB Centre, Department of Paediatrics and Child Health, Faculty of Medicine and Health Sciences, Stellenbosch University, Cape Town, South Africa;; ^2^ School of Public Health, Faculty of Medicine and Health, The University of Sydney, Sydney, NSW, Australia;; ^3^ THINK Tuberculosis and HIV Investigative Network (RF) NPC, Durban, South Africa;; ^4^ Global Alliance for TB Drug Development (TB Alliance), New York, NY, USA;; ^5^ Department of Pediatrics, University of Wisconsin-Madison School of Medicine and Public Health, Madison, WI, USA.

**Keywords:** rifampicin-resistant, moxifloxacin, linezolid, acceptability, palatability, children

## Abstract

**BACKGROUND:**

Moxifloxacin (MFX) and linezolid (LZD) are key components of rifampicin-resistant TB treatment regimens. Currently, available dispersible tablet formulations of both drugs have poor palatability in children. We evaluated children’s preferences for more child-friendly formulations from two generic manufacturers.

**METHODS:**

This was a randomised, cross-sectional ‘swish-and-spit’ taste panel study at two sites in South Africa. Each manufacturer created three flavour-blend variants for each drug. Healthy child volunteers 5–17 years old were sampled stratified by age, sex, and ethnic group and completed a preference rank-ordering and five acceptability 5-point Likert scales. We explored the blends’ acceptability using summary, comparative, and ranking statistics.

**RESULTS:**

Ninety-seven and 96 children contributed data for the MFX and LZD drug blends, respectively. For both manufacturers’ MFX blends, the Friedman test showed children had a statistically significant preference for novel options over the Existing blend (Q(2) = 24,937; *P* < 0.001, and Q(2) = 21.213; *P* < 0.001, respectively). Even the most preferred MFX blend had sub-optimal acceptability, especially for one manufacturer. Children did not have a clear preference for both manufacturers’ LZD blends. These findings were not influenced by age, sex, or ethnic group.

**CONCLUSION:**

Children across a broad age spectrum can provide meaningful input on the palatability preferences of TB drug formulations. Novel MFX formulation blends were recommended for development, but acceptability remains suboptimal.

Rifampicin-resistant TB (RR-TB) is *Mycobacterium tuberculosis* resistant to at least rifampicin, one of four first-line drugs. Model-based estimates suggest there are at least 25,000 to 32,000 new cases of RR- and multidrug-resistant TB (MDR-TB) (resistant to at least rifampicin and isoniazid) among children 0–14 years old each year globally.^1,2^

Treatment outcomes among children treated for RR-TB are better than in adults, with meta-analysis showing 78% successfully treated.^3^ This remains below targets of at least 90% successfully treated, so ensuring access to better, safer, shorter, and more child-friendly treatment for all children with RR-TB is a high priority.^4^

Moxifloxacin (MFX) and linezolid (LZD) are key components of current routine RR-TB treatment regimens and WHO ‘Group A’ drugs, and they will continue to be part of regimens going forward. Children’s access to suitable formulations has been limited due to a lack of safety, pharmacokinetic, and dosing data and a lack of investment in developing palatable formulations. MFX has a very bitter taste with extremely poor palatability, and LZD’s palatability for children is unknown, described only in one small study as similarly unpalatable.^5^

Acceptability is the overall suitability of a formulation, including the dose volume or size and palatability.^6^ Palatability is the acceptance of taste, smell, volume or size, and texture of an oral medication.^7^ Palatability is extremely important in younger children (<5 years old) who often drink suspensions made from crushed or dispersible tablets.^8^ Poor palatability is a risk to both dosing accuracy, as children will spit up unpalatable medication, and treatment completion/adherence as caregivers discontinue to avoid the trauma of administration.^9–11^ Minimising these treatment-associated morbidities is critical to enable the effective use of these priority drugs in children.

Few studies of acceptability are done on children in resource-limited settings.^5,11^ Genetic differences in bitter-type receptors^12,13^ may influence palatability. Food choice and taste preference are highly complex human traits dependent on cultural, social, environmental, genetic, and biological factors.^14–17^

We report on the ChilPref ML study, which aimed to inform which versions of new child-friendly formulation blends of MFX and LZD are preferred by children to inform taste optimisation of commercially available dispersible tablet formulations from two generic manufacturers.

## METHODS

### Study design

ChilPref ML was a cross-sectional ‘swish-and-spit’ taste panel evaluation conducted at two sites in South Africa. The ‘swish-and-spit’ taste panel is an established approach for paediatric formulation evaluation where healthy volunteers taste, spit out, and then report on the formulations’ relative palatability. No medicines are ingested.

### Setting and study population

The study was conducted in Cape Town (Western Cape Province) and uMgungundlovu and eThekwini (KwaZulu-Natal). Both provinces have high RR-TB burden, and current guidelines recommend using MFX and LZD for the treatment of RR-TB in children. Together, these districts’ populations have a variety of usual diets, proxied by rural/urban location and ethnic identity.

### Eligibility

We enrolled healthy child volunteers, 5–17 years old, from high TB burden communities at these sites. Children were ineligible if they were living with someone current TB disease (suspected or confirmed), were a current contact with a TB patient, were undergoing dental care or treatment in the past 14 days, had used mouthwash within 24 hours of the study visit, had smoked in the preceding 48 hours, had known drug allergies or history of hypersensitivity to antimicrobial agents, or intolerance to saltine crackers, or had any other condition that could have influenced taste or smell sensation, or inability to perform the ranking activity. All participants and caregivers were informed of inclusion/exclusion criteria. Screening for enrolment was based on self-report supervised by the study coordinator, a trained nurse.

### Sampling and recruitment

We aimed to recruit 96 children per taste panel to achieve a minimum of 72 evaluable participants. Evaluable participants were those who completed taste evaluations for all three blends of a given drug from one of the drug manufacturers. The 96 intended participants were stratified by gender, site, and age group: 5–8 years, 9–12 years, and 13–17 years. The study was not powered to detect differences between these factors; stratification by gender, site, and age group was a sampling strategy to support the transferability of the findings.

### Formulation flavour blend

Table 1 describes the flavour blends of MFX and LZD formulations from Macleods Pharmaceuticals (Mumbai, India) and Micro Labs Limited (Bangalore, India). Each pharmaceutical manufacturer supplied three different formulation versions (blends) of MFX and three of LZD; therefore, six versions of each drug in total were evaluated. One version of each drug from each manufacturer was the exact content of a WHO-prequalified approved or soon-to-be-approved dispersible tablet, including the active pharmaceutical ingredient (API) and all excipients and flavours. Both manufacturers had followed routine internal processes to select flavours for this ‘Existing blend’ that they believed suited to children. The other two blends contained the same API and excipients but with different flavours selected in discussion with the project team informed by prior research on children’s TB drug taste preferences (Table 1).^11^ Flavour changes were only made with additives that are Generally Recognised as Safe (GRAS) by the FDA and thus exempt from the usual detailed evaluation required of food additives.

**Table 1.** Existing and novel blends of moxifloxacin and linezolid from Macleods Pharmaceutical (Mumbai, India) and Micro Labs (Bangalore, India) evaluated in the ChilPref-ML study.

Drug	Study label	Flavour	Existing or novel blend
Moxifloxacin			
Macleods Pharmaceuticals			
	A1	Orange	Novel blend 1
	A2	Lemon and peppermint	Existing blend
	A3	Bitter masker	Novel blend 2
Micro Labs			
	B1	Pineapple	Existing blend
	B2	Strawberry and raspberry	Novel blend 2
	B3	Tutti frutti	Novel blend 1
Linezolid			
Macleods Pharmaceuticals			
	C1	Butterscotch and peppermint	Novel blend 2
	C2	Orange and peppermint	Novel blend 1
	C3	Peppermint and strawberry	Existing blend
Micro Labs			
	D1	Lemon and peppermint	Novel blend 1
	D2	Pineapple and peppermint	Novel blend 2
	D3	Orange and peppermint	Existing blend

### Data collection

Participants completed the MFX taste panel and the LZD taste panel on separate days – i.e., they tasted six blends over approximately 60 minutes per day. Data were collected between March–June 2022. For the taste panel evaluation, participants would taste a formulation blend, rank each blend in order of preference to the others and complete a brief palatability questionnaire. The rank-ordering was iterative, i.e., after tasting the second blend, the participant was asked if it tasted better or worse than the first blend and so on. The end of the process was for the researcher to confirm that the order of preferences indicated on the table reflected the participants’ perspective, then capture that rank order on an electronic data capture form. The rank ordering was within each manufacturer’s three blends. The flavour blends were formulated as dispersible tablets that were dispersed in water. Each tablet was diluted with a fixed volume of 10 mL of water, which participants swished in their mouths and then spat out after 3–5 seconds without ingesting the mixture. To account for any lingering taste or interaction effects between blends, the order of tasting blends was randomly assigned for each participant. Between formulation tastings, participants were asked to drink water and eat a taste-neutral food that lasted approximately 5–10 minutes.

After each tasting, the participant completed five 5-point Likert-scale type items on taste response aspects to assess the palatability of each blend: taste (I like the way that tasted!), smell (That smelled good!), after-taste (The after-taste was really nice!), emotional response (That taste made me feel good!), and overall taste (Overall, that tasted great!). Each Likert response category (Strongly Agree, Agree, Neutral, Disagree, and Strongly Disagree) included these words and a visual hedonic scale (stylised ‘smiley-faces’) corresponding to the sentiment. The scale was administered by the researcher while the participant was drinking water and eating a taste neutral food (cracker). Responses were entered into an electronic data capture service. Participants then tasted the next blend and repeated this process until all six blends had been tasted.

### Randomisation

The formulations were presented in a balanced, randomised order as determined by a Williams design where each formulation is represented once in each sequence, and each treatment only followed another treatment an equal number of times. Six formulation sequences derived from the Williams design were used since three formulations were evaluated simultaneously. To ensure that the order of drugs was randomly assigned, 12 sequences were utilised so that each drug manufacturer had an equal chance of being tasted first. This effectively meant that the order of tasting the three blends of a drug from each manufacturer was randomised, and the order of the manufacturer was also randomised. Each child was randomised to one of 12 sequences using blocked randomisation, and each child’s sequence was revealed at the time of the first formulation tasting via an online randomisation service (Sealed Envelope). Both the participants and the data collection teams were blinded to the intended flavours of the blends and whether these were the manufacturers’ Existing or novel blends.

### Statistical analysis

All quantitative data were analysed using Stata Special Edition v16.1 (Stata Corp, College Station, TX, USA).^19^ Age, gender, and ethnicity (as a proxy for usual diet) were summarised in aggregate and by study site, using descriptive statistics. The taste preference rankings for each blend were described separately for MFX and LZD by the manufacturer using summary statistics and were disaggregated by age and ethnicity. The Friedman test was used to determine if the taste preference rankings differed statistically by blend. If a taste preference statistical difference was identified amongst the MFX or the LZD formulations, then post hoc testing using the Dunn-Bonferroni test for multiple comparisons was done.

The Likert data palatability characteristics were described separately for MFX and LZD and were disaggregated by age and ethnicity. The Likert data collected from the 5-point facial hedonic scales for palatability characteristics were described overall and by drug manufacturers using summary statistics that were dichotomised as follows: Strongly Agree/Agree/Neutral versus Disagree/Strongly Disagree. The dichotomised hedonic scale items for drug formulations were compared using the Cochrane’s *Q* test. When relevant, post hoc testing was done using the McNemar test with the Benjamini-Hochberg method to adjust for multiple comparisons.

The Likert data were combined to form an overall taste experience score of the participants’ palatability preferences. The overall taste experience score was calculated by summing each item from the preferences hedonic scale as coded in the database. When calculating the overall taste experience score, the values used were Strongly Agree = 1, Agree = 2, Neutral = 3, Disagree = 4, and Strongly Disagree = 5. Lower scores represent a better overall taste experience in comparison to higher scores. The minimum and maximum scores a participant could have for their overall taste experience score were 5 and 25, respectively. A one-way repeated measures analysis of variance was used to compare the overall taste experience scores of the formulation blends for each drug. If there were statistically significant differences in the overall taste experience score, post hoc testing was done using Tukey’s method for multiple comparisons.

For whatever reason, participants who did not complete or only partially completed the evaluation of the three blends from one drug manufacturer were excluded from the analysis of those formulation evaluations that they did not complete.

### Ethics statement and details of informed consent

The study was approved by the Health Research Ethics Committee at Stellenbosch University, Stellenbosch, South Africa (N21/06/057) and the WHO Ethical Review Committee. Caregivers of all participants signed written informed consent for their child’s participation. Children aged 7–17 years completed written assent, while children aged 5–6 years provided verbal assent.

## RESULTS

Respectively, 97 and 96 participants were randomised, enrolled for the study and contributed data for MFX and LZD. Participants’ demographic characteristics are displayed in Table 2 in aggregate and by study site. Table 3 shows the overall taste preference rankings for MFX and LZD formulations. A lower rank represents a better overall taste preference than a higher rank. The most preferred drug formulation received a value of 1, the following preferred drug formulation received a value of 2, and the least preferred drug formulation received a value of 3.

**Table 2.** Demographic characteristics at randomisation by study site and drug.

	All participants	DTTC	THINK
	(*n* = 97)	(*n* = 49)	(*n* = 48)
	*n* (%)	*n* (%)	*n* (%)
Moxifloxacin			
Male sex	49 (50.5)	24 (49.0)	25 (52.1)
Ethnicity*			
Black, urban	29 (29.9)	24 (49.0)	5 (10.4)
Black, rural	18 (18.6)	0 (0.0)	18 (37.5)
Coloured	25 (25.8)	25 (51.0)	0 (0.0)
Indian	25 (25.5)	0 (0.0)	25 (52.1)
Age, years, median [IQR]	10.8 [8.0–13.0]	10.8 [7.0–13.0]	10.9 [8.0–13.5]
Age category, years			
5–8	32 (33.0)	16 (32.7)	16 (33.3)
9–12	33 (34.0)	17 (34.7)	16 (33.3)
13–17	32 (33.0)	16 (32.7)	16 (33.3)
Linezolid	All participants (*n* = 96)	DTTC (*n* = 48)	THINK (*n* = 48)
Male sex	32 (33.0)	16 (32.7)	16 (33.3)
Ethnicity*			
Black, urban	27 (28.1)	24 (50.0)	3 (6.3)
Black, rural	21 (21.9)	0 (0.0)	21 (43.8)
Coloured	24 (25.0)	24 (50.0)	0 (0.0)
Indian	24 (25.0)	0 (0.0)	24 (50.0)
Age, years, median [IQR]	10.9 [7.0–14.0]	10.9 [7.0–14.0]	10.9 [7.5–13.5]
Age category, years			
5–8	32 (33.3)	16 (33.3)	16 (33.3)
9–12	33 (34.4)	17 (35.4)	16 (33.3)
13–17	31 (32.3)	15 (31.3)	16 (33.3)

* Ethnicity is used as a proxy for the usual diet. In South Africa, people who self-identify as ‘Coloured’ are a distinct cultural group with a diverse history, including so-called ‘mixed-race’, indigenous Khoi and San peoples, and Southeast Asian slaves.

DTTC = Desmond Tutu TB Centre; THINK = TB and HIV Investigative Network; IQR = interquartile range.

**Table 3.** Mean rank comparison for Macleods and Micro Labs blends for moxifloxacin and linezolid.

	Rank sum	Rank^*^	Friedman’s F (*P*-value)
		Mean ± SD	
Moxifloxacin			
Overall, which Macleods moxifloxacin formulation did the child prefer to take? (*n* = 96)			
A1: Novel blend 1	180	1.88 ± 0.81	
A2: Existing blend	231	2.41 ± 0.69	
A3: Novel blend 2	165	1.72 ± 0.79	24.937 (<0.001)^†^
Overall, which Micro Labs moxifloxacin formulation did the child prefer to take? (*n* = 94)			
B1: Existing blend	224	2.38 ± 0.79	
B2: Novel blend 2	165	1.76 ± 0.79	
B3: Novel blend 1	175	1.86 ± 0.74	21.213 (<0.001)^‡^
Linezolid			
Overall, which Macleods linezolid formulation did the child prefer to take? (*n* = 95)			
C1: Novel blend 2	190	2.00 ± 0.86	
C2: Novel blend 1	186	1.96 ± 0.81	
C3: Existing blend	194	2.04 ± 0.78	0.337 (0.884)
Overall, which Micro Labs linezolid formulation did the child prefer to take? (*n* = 95)			
D1: Novel blend 1	195	2.05 ± 0.78	
D2: Novel blend 2	187	1.97 ± 0.86	
D3: Existing blend	188	1.98 ± 0.82	0.400 (0.819)

* The rank values for the overall taste preference are: 1 = most preferred; 2 = moderately preferred; 3 = least preferred. A lower rank represents a better overall taste preference in comparison to a higher rank.

† Post-hoc testing using the Dunn-Bonferroni test for multiple comparisons indicates that the mean ranks were statistically significant between drug formulations A1 (Novel blend 1) and A2 (Existing blend) (*P* < 0.001) and drug formulations A2 (Existing blend) and A3 (Novel blend 2) (*P* < 0.001). There were no differences between drug formulations A1 (Novel blend 1) and A3 (Novel blend 2) (*P* = 0.279).

‡ Post-hoc testing using the Dunn-Bonferroni test for multiple comparisons indicates that the mean ranks were statistically significant between drug formulations B1 (Existing blend) and B2 (Novel blend 2) (*P* < 0.001) and B1 (Existing blend) and B3 (Novel blend 1) (*P* < 0.001). There were no differences between drug formulations B2 (Novel blend 2) and B3 (Novel blend 1) (*P* = 0.559).

SD = standard deviation.

For the MFX blends prepared by Macleods, there was a statistically significant difference in taste preference rankings between A1, A2 and A3: Q(2) = 24,937, *P* < 0.001 – with the ‘Bitter masker’ flavour blend most preferred. Post hoc testing indicated the mean ranks were statistically significant between drug formulations Novel blend 1 and Existing blend (*P* < 0.001), and Existing blend and Novel blend 2 (*P* < 0.001).

For the MFX blends prepared by Micro Labs, there was a statistically significant difference in taste preference rankings between the B1, B2 and B3: Q(2) = 21.213, *P* < 0.001 – with the ‘Strawberry & Raspberry’ flavour blend most preferred. Post-hoc testing indicated the mean ranks were statistically significant between drug formulation blends Existing blend and Novel blend 2 (*P* < 0.001) and Existing blend and Novel blend 1 (*P* < 0.001).

Analyses for both manufacturers stratified by age and ethnicity showed similar results and are shown in Supplementary Tables S1–S4.

There were no significant differences in taste preferences for either Macleods’ or Micro Labs’ LZD blends. Similarly, there were no trends in preferences for LZD blends when disaggregated by age or ethnicity (Supplementary Tables S5–S8). Table 4 displays the responses to the palatability comparisons for the MFX blends. Post-hoc testing indicated Macloeds’ Existing blend had significantly more unfavourable palatability responses to ‘I liked the way that tasted!’ ‘That smelled good!’ and ‘Overall, that tasted great!’ in comparison to Novel blend 1 (*P* = 0.007, *P* < 0.001 and *P* = 0.036, respectively) and Novel blend 2 (*P* = 0.003, *P* < 0.001 and *P* < 0.001, respectively). Post hoc testing indicated that Macleods’ Existing blend had an overall taste experience score that was significantly higher (worse) in comparison to Novel Blend 1 (*P* = 0.006) and Novel Blend 2 (*P* < 0.001).

**Table 4.** Palatability of Macleods Pharmaceutical (Mumbai, India) and Micro Labs (Bangalore, India) existing and novel moxifloxacin blends.

	Macleods Pharmaceuticals				Micro Labs			
	A1	A2	A3	*P*-value	B1	B2	B3	*P*-value
	Novel blend 1	Existing blend	Novel blend 2		Existing blend	Novel blend 2	Novel blend 1	
	(*n* = 97)	(*n* = 97)	(*n* = 97)		(*n* = 97)	(*n* = 97)	(*n* = 97)	
	*n* (%)	*n* (%)	*n* (%)		*n* (%)	*n* (%)	*n* (%)	
I liked the way that tasted!								
Strongly agree or agree or neutral	68 (70.1)	51 (52.6)	68 (70.1)		27 (27.8)	58 (59.8)	48 (49.5)	
Disagree or strongly disagree	29 (29.9)	46 (47.4)	29 (29.9)	0.002^*^	70 (72.2)	39 (40.2)	49 (50.5)	<0.001^†^
That smelled good!								
Strongly agree or agree or neutral	89 (91.8)	70 (72.2)	89 (91.8)		82 (84.5)	86 (88.7)	86 (88.7)	
Disagree or strongly disagree	8 (8.2)	27 (27.8)	8 (8.2)	<0.001^‡^	15 (15.5)	11 (11.3)	11 (11.3)	0.576
The after-taste was really nice!								
Strongly agree or agree or neutral	46 (47.4)	44 (45.4)	57 (58.8)		25 (25.8)	38 (39.2)	38 (39.2)	
Disagree or strongly disagree	51 (52.6)	53 (54.6)	40 (41.2)	0.050	72 (74.2)	59 (60.8)	59 (60.8)	0.036^§^
That taste made me feel good!								
Strongly agree or agree or neutral	60 (61.9)	52 (53.6)	65 (67.0)		32 (33.0)	53 (54.6)	45 (46.4)	
Disagree or strongly disagree	37 (38.1)	45 (46.4)	32 (33.0)	0.080	65 (67.0)	44 (45.4)	52 (53.6)	0.001^¶^
Overall, that tasted great!								
Strongly agree or agree or neutral	54 (55.7)	41 (42.3)	63 (64.9)		27 (27.8)	51 (52.6)	46 (47.4)	
Disagree or strongly disagree	43 (44.3)	56 (57.7)	34 (35.1)	<0.001^#^	70 (72.2)	46 (47.4)	51 (52.6)	<0.001^**^
Overall taste experience score^††^								
Mean ± SD	13.6 ± 5.7	15.6 ± 5.8	12.6 ± 5.7		17.7 ± 5.0	14.9 ± 5.1	15.8 ± 5.3	
[IQR]	[8.0–19.0]	[11.0–20.0]	[8.0–17.0]	<0.001^‡‡^	[16.0–21.0]	[11.0–19.0]	[12.0–20.0]	<0.001^§§^

* Post-hoc McNemar tests using the Benjamini-Hochberg adjustment for multiple comparisons indicated that responses to the question ‘I like the way that tasted!’ were statistically different between drug formulations A1 (Novel blend 1) and A2 (Existing blend) (*P* = 0.007), A2 (Existing blend) and A3 (Novel blend 2) (*P* = 0.003); there were no differences between A1 (Novel blend 1) and A3 (Novel blend 2) (*P* = 1.000).

† Post-hoc McNemar tests using the Benjamini-Hochberg adjustment for multiple comparisons indicated that responses to the question ‘I like the way that tasted!’ were statistically different between drug formulations B1 (Existing blend) and B2 (Novel blend 2) (*P* < 0.001) and B1 (Existing blend) and B3 (Novel blend 1) (*P* = 0.001); there were no differences between B2 (Novel blend 2) and B3 (Novel blend 1) (*P* = 0.123).

‡ Post-hoc McNemar tests using the Benjamini-Hochberg adjustment for multiple comparisons indicated that responses to the question ‘That smelled good!’ were statistically different between drug formulations A1 (Novel blend 1) and A2 (Existing blend) (*P* < 0.001) and A2 (Existing blend) and A3 (Novel blend 2) (*P* < 0.001) while there were no differences between A1 (Novel blend 1) and A3 (Novel blend 2) (*P* = 1.000).

§ Post-hoc McNemar tests using the Benjamini-Hochberg adjustment for multiple comparisons indicated that responses to the question ‘The after-taste was really nice!’ were statistically different between drug formulations B1 (Existing blend) and B3 (Novel blend 1) (0.036); there were no differences between B1 (Existing blend) and B2 (Novel blend 2) (p=0.071) and between B2 (Novel blend 2) and B3 (Novel blend 1) (*P* = 1.000).

¶ Post-hoc McNemar tests using the Benjamini-Hochberg adjustment for multiple comparisons indicated that responses to the question ‘That taste made me feel good!’ were statistically different between drug formulations B1 (Existing blend) and B2 (Novel blend 2) (*P* < 0.001) and B1 (Existing blend) and B3 (Novel blend 1) (*P* = 0.042); there were no differences between B2 (Novel blend 2) and B3 (Novel blend 1) (*P* = 0.170).

# Post-hoc McNemar tests using the Benjamini-Hochberg adjustment for multiple comparisons indicated that responses to the question ‘Overall, that tasted great!’ were statistically different between drug formulations A1 (Novel blend 1) and A2 (Existing blend) (*P* = 0.036) and A2 (Existing blend) and A3 (Novel blend 2) (*P* < 0.001) while there were no differences between A1 (Novel blend 1) and A3 (Novel blend 2) (*P* = 0.106).

** Post-hoc McNemar tests using the Benjamini-Hochberg adjustment for multiple comparisons indicated that responses to the question ‘Overall, that tasted great!’ were statistically different between drug formulations B1 (Existing blend) and B2 (Novel blend 2) (*P* < 0.001) and B1 (Existing blend) and B3 (Novel blend 1) (*P* = 0.004); there were no differences between B2 (Novel blend 2) and B3 (Novel blend 1) (*P* = 0.435).

†† Overall taste experience was calculated by summing each of the items from the preferences hedonic scale as coded in the database for each of the drug formulations. The minimum and maximum scores that a participant could have was 5 and 25, respectively. The values for the hedonic scare are: Strongly agree = 1, Agree = 2, Neutral = 3, Disagree = 4, Strongly Disagree = 5. Lower scores represent a better overall taste experience in comparison to higher scores.

‡‡ Post hoc comparisons using Tukey’s method for multiple comparisons indicated that the overall taste experience score was significantly different between drug formulations A1 (Novel blend 1) and A2 (Existing blend) (*P* = 0.006) and formulations A2 (Existing blend) and A3 (Novel blend 2) (*P* < 0.001). There were no statistically significant differences between drug formulation A1 (Novel blend 1) and A3 (Novel blend 2) (*P* = 0.209).

§§ Post hoc comparisons using Tukey’s method for multiple comparisons indicated that overall taste experience score was significantly different between drug formulations B1 (Existing blend) and B2 (Novel blend 2) (*P* < 0.001) and formulations B1 (Existing blend) and B3 (Novel blend 1) (*P* = 0.003). There were no statistically significant differences between drug formulation B2 (Novel blend 2) and B3 (Novel blend 1) (*P* = 0.284).

Analyses of the overall taste experience scores stratified by age and ethnicity showed similar results, as shown in Supplementary Table S9. Post-hoc testing indicated Micro-Labs MFX Existing blend had significantly more unfavourable palatability responses to ‘I liked the way that tasted!’, ‘That taste made me feel good’ and ‘Overall, that tasted great!’ in comparison to Novel blend 2 (*P* < 0.001, *P* < 0.001 and *P* < 0.001, respectively) and Novel blend 1 (*P* = 0.001, *P* = 0.042 and *P* = 0.004, respectively). Post hoc testing indicated that Micro-Lab’s Existing blend had an overall taste experience score that was significantly higher (worse) in comparison to Novel Blend 2 (p < 0.001) and Novel Blend 1 (*P* = 0.003). Analyses of the overall taste experience scores stratified by age and ethnicity showed similar results, as shown in Supplementary Table S10. Table 5 displays the palatability comparisons of the LZD formulations.

**Table 5.** Palatability of Macleods Pharmaceutical (Mumbai, India) and Micro Labs (Bangalore, India) existing and novel linezolid blends.

	Macleods Pharmaceuticals				Micro Labs			
	C1	C2	C3	*P*-value	D1	D2	D3	*P*-value
	Novel blend 2	Novel blend 1	Existing blend		Novel blend 1	Novel blend 2	Existing blend	
	(*n* = 96)	(*n* = 95)	(*n* = 96)		(*n* = 96)	(*n* = 96)	(*n* = 95)	
	*n* (%)	*n* (%)	*n* (%)		*n* (%)	*n* (%)	*n* (%)	
I liked the way that tasted!								
Strongly agree or agree or neutral	78 (81.3)	68 (71.6)	66 (68.8)		68 (70.8)	78 (81.3)	68 (70.8)	
Disagree or strongly disagree	18 (18.8)	27 (28.4)	30 (31.3)	0.042*	28 (29.2)	18 (18.8)	28 (29.2)	0.082
That smelled good!								
Strongly agree or agree or neutral	88 (91.7)	88 (92.6)	85 (88.5)		86 (89.6)	85 (88.5)	88 (91.7)	
Disagree or strongly disagree	8 (8.3)	7 (7.4)	11 (11.5)	0.486	10 (10.4)	11 (11.5)	8 (8.3)	0.705
The after-taste was really nice!								
Strongly agree or agree or neutral	66 (68.8)	63 (66.3)	58 (60.4)		52 (54.2)	58 (60.4)	58 (60.4)	
Disagree or strongly disagree	30 (31.3)	32 (33.7)	38 (39.6)	0.412	44 (45.8)	38 (39.6)	38 (39.6)	0.457
That taste made me feel good!								
Strongly agree or agree or neutral	73 (76.0)	65 (68.4)	63 (65.6)		64 (66.7)	68 (70.8)	59 (62.1)	
Disagree or strongly disagree	23 (24.0)	30 (31.6)	33 (34.4)	0.159	32 (33.3)	28 (29.2)	36 (37.9)^§^	0.294
Overall, that tasted great!								
Strongly agree or agree or neutral	75 (78.1)	69 (72.6)	64 (66.7)		63 (65.6)	70 (72.9)	69 (71.9)	
Disagree or strongly disagree	21 (21.9)	26 (27.4)	32 (33.3)	0.196	33 (34.4)	26 (27.1)	27 (28.1)	0.376
Overall taste experience score^†^								
Mean ± SD	11.7 ± 4.6	12.6 ± 5.2	13.2 ± 5.4		13.1 ± 5.2	12.3 ± 5.0	13.0 ± 5.4	
[IQR]	[8.0–15.0]	[8.0–17.0]	[8.0–18.0]	0.029^‡^	[9.0–17.5]	[8.0–16.0]	[8.0–17.0]	0.415

* Post-hoc McNemar tests using the Benjamini-Hochberg adjustment for multiple comparisons indicate that responses to the question, ‘I like the way that tasted!’ were statistically significant between drug formulations C1 (Novel blend 2) and C3 (Existing blend) (*P* = 0.042) while there were no differences between C1 (Novel blend 2) and C2 (Novel blend 1) (*P* = 0.142) and C2 (Novel blend 1) and C3 (Existing blend) (*P* = 0.513).

† Overall taste experience was calculated by summing each of the items from the preferences hedonic scale as coded in the database for each of the drug formulations. The minimum and maximum scores that a participant could have was 5 and 25, respectively. The values for the hedonic scare are: Strongly agree = 1, Agree = 2, Neutral = 3, Disagree = 4, Strongly Disagree = 5. Lower scores represent a better overall taste experience in comparison to higher scores.

‡ Post-hoc comparisons using Tukey’s method for multiple comparisons indicated that overall taste experience score was significantly different between formulation C1 (Novel blend 2) and C3 (Existing blend) (*P* = 0.024). There were no statistically significant differences between drug formulation C1 (Novel blend 2) and C2 (Novel blend 1) (*P* = 0.203) and drug formulation C2 (Novel blend 1) and C3 (Existing blend) (*P* = 0.620).

§ (*n* = 95) since one participant did not provide a response to the item ‘That taste made me feel good!’ for drug formulation D3 (Existing blend).

For the Macleods LZD blends, post-hoc analysis indicated Existing blend had a higher proportion of participants with a favourable response to ‘I liked the way that tasted!’ in comparison to Novel blend 2 (*P* = 0.043). Post-hoc testing indicated that Macleods’ Existing blend had an overall taste experience score that was significantly higher (worse) in comparison to Novel Blend 2 (*P* = 0.024). Analyses of the overall taste experience scores stratified by age and ethnicity showed similar results (Supplementary Tables S11 and S12). Combining these analyses, we concluded that for MFX, Macleods’ Novel Blend 2 and Micro Labs’ Novel Blend 2 were the most suitable blends to carry forward for commercial development. For LZD, we concluded that there was insufficient evidence to warrant the cost to change from either manufacturer’s Existing blends.

## DISCUSSION

We identified novel flavour blends of MFX for both generics manufacturers that were statistically significantly preferred over their Existing formulation blends by children from a high TB burden setting. These results were reported to both generic manufacturers, who are updating their Existing formulations accordingly. This will result in commercially available MFX-dispersible tablets with improved palatability. The study confirms anecdotal reports that the Existing commercially available MFX formulations are very poorly palatable, with 57.7% and 72.2% of participants in our study disliking the overall taste of these Existing formulation blends. Previous data have shown the importance of medication taste to children and their caregivers and the potential treatment-related harms caused by poor palatability on adherence, dosing and treatment experiences.^8,9^ The study demonstrates that relatively simple and inexpensive changes in MFX formulation blend had the potential to significantly improve palatability. For LZD, we found no statistical preference by flavour blend for either manufacturer and, therefore, no reason to recommend a change from the Existing formulation.

The overall palatability of all blends, even the preferred ones, was sub-optimal. This was especially true for moxifloxacin, for which 35.1% and 47.4% of participants still did not like the taste of the most preferred blends evaluated in the study. There may have been additional scope to further improve the formulations’ palatability by adjusting excipients other than the flavouring agent or alternative flavouring agents. Better taste masking may be difficult, especially for MFX, known to be a bitter compound. Regardless, it is important to be aware that some children may still dislike these improved formulations so that decisions about regimen choice can be made and caregivers and children taking these formulations can be appropriately supported. The study shows that the swish-and-spit taste panel design, including the rank ordering and palatability scales, was feasible for young children and adolescents. Children across the included age spectrum were able to understand the process, safely and successfully follow the instructions and share intelligible preferences. This is the first application of this approach to TB drug formulation evaluation in children. Children may have different taste preferences than adults,^8,18,19^ and the end users of these formulations should have the opportunity to share their preferences directly.

### Limitations

Our study was implemented in only two South African provinces, and its generalizability to other populations is uncertain. To mitigate this, the study deliberately included a diverse population within South Africa, where taste receptor genetics and usual diet may differ. Age, gender, and ethnicity did not influence preferences or palatability characteristics, though the analysis was not powered for these secondary analyses. These are still important to consider, and future studies should target populations diverse in these characteristics until their impact on palatability has been more clearly defined. An additional limitation is that the pre-taste panel development of formulation blends was limited to minor tweaking of flavour and could be further improved.

## CONCLUSION

This innovative study design and approach can be replicated for other medications, including further novel LZD and MFX formulation blends. Children’s reported preferences should be included earlier in the development process, prior to formulations being finalised, licensed for use, and commercially available. These data may also be relevant for adults to improve the acceptability of TB treatment and adherence. Better, more child-friendly formulations are an important public health good, and investment in similar work for Existing and future drugs will improve experiences and outcomes for children with TB and other diseases.

## Supplementary Material


