# Supplementary material for: Children’s preferences among six novel moxifloxacin and linezolid-dispersible tablet formulations
Source: IJTLD Open. 2025 Apr 9;2(4):208–16. doi: 10.5588/ijtldopen.24.0546 (PMC11984522; doi:10.5588/ijtldopen.24.0546)
Supplement: Supplementary file 1 [file ijtldopen24-0546_supplementarydata1.docx]

# **Children’s preferences among six novel moxifloxacin and linezolid-dispersible tablet formulations**

**Supplementary Data**

**S1: Mean Rank Comparison for Macleods Blends for Moxifloxacin by Age Group**

|  | **5 – 8 years old** | | | **9 – 12 years old** | | | **13 – 17 years old** | | |
| --- | --- | --- | --- | --- | --- | --- | --- | --- | --- |
|  | Rank Sum | Mean Rank  (SD) | Friedman’s F (p-value) | Rank Sum | Mean Rank  (SD) | Friedman’s F (p-value) | Rank Sum | Mean Rank  (SD) | Friedman’s F (p-value) |
|  |  |  |  |  |  |  |  |  |  |
| Overall, which Manufacturer 1 moxifloxacin formulation did the child prefer to take? |  |  |  |  |  |  |  |  |  |
| A1 (Novel blend 1) | 56 | 1.75 (0.76) |  | 65 | 2.03 (0.82) |  | 59 | 1.84 (0.85) |  |
| A2 (Existing blend) | 74 | 2.31 (0.74) |  | 81 | 2.53 (0.62) |  | 76 | 2.38 (0.71) |  |
| A3 (Novel blend 2) | 62 | 1.94 (0.88) | 5.25 (0.072) | 46 | 1.44 (0.62) | 19.19 (<0.001)* | 57 | 1.78 (0.79) | 6.81 (0.033)± |
|  |  |  | (n=32) |  |  | (n=32) |  |  | (n=32) |
|  |  |  |  |  |  |  |  |  |  |

* Post-hoc testing using the Dunn-Bonferroni test for multiple comparisons indicates the mean ranks were statistically significant between drug formulations A1 (Novel blend 1) and A2 (Existing blend) (p=0.045), drug formulations A1 (Novel blend 1) and A3 (Novel blend 2) (p=0.011) and drug formulations A2 (Existing blend) and A3 (Novel blend 2) (p<0.001).

± Post-hoc testing using the Dunn-Bonferroni test for multiple comparisons indicates the mean ranks were statistically significant between drug formulations A1 (Novel blend 1) and A2 (Existing blend) (p=0.029) and drug formulations A2 (Existing blend) and A3 (Novel blend 2) (p=0.011). There were no differences between drug formulations A1 (Novel blend 1) and A3 (Novel blend 2) (p=1.000).

**S2: Mean Rank Comparison for Macleods Blends for Moxifloxacin by Ethnicity**

|  | **Black urban** | | | **Black rural** | | |
| --- | --- | --- | --- | --- | --- | --- |
|  | Rank Sum | Mean Rank  (SD) | Friedman’s F (p-value) | Rank Sum | Mean Rank  (SD) | Friedman’s F (p-value) |
|  |  |  |  |  |  |  |
| Overall, which Macleods moxifloxacin formulation did the child prefer to take? |  |  |  |  |  |  |
| A1 (Novel blend 1) | 53 | 1.83 (0.89) |  | 30 | 1.67 (0.77) |  |
| A2 (Existing blend) | 72 | 2.48 (0.51) |  | 43 | 2.39 (0.78) |  |
| A3 (Novel blend 2) | 49 | 1.69 (0.81) | 10.414 (0.006)* | 35 | 1.94 (0.80) | 4.778 (0.092) |
|  |  |  | (n=29) |  |  | (n=18) |
|  |  |  |  |  |  |  |

|  | **Coloured** | | | **Indian** | | |
| --- | --- | --- | --- | --- | --- | --- |
|  | Rank Sum | Mean Rank  (SD) | Friedman’s F (p-value) | Rank Sum | Mean Rank  (SD) | Friedman’s F (p-value) |
|  |  |  |  |  |  |  |
| Overall, which Macleods moxifloxacin formulation did the child prefer to take? |  |  |  |  |  |  |
| A1 (Novel blend 1) | 49 | 2.04 (0.81) |  | 48 | 1.92 (0.76) |  |
| A2 (Existing blend) | 55 | 2.29 (0.75) |  | 61 | 2.44 (0.77) |  |
| A3 (Novel blend 2) | 40 | 1.67 (0.82) | 4.750 (0.093) | 41 | 1.64 (0.76) | 8.240 (0.016)± |
|  |  |  | (n=24) |  |  | (n=25) |
|  |  |  |  |  |  |  |

* Post-hoc testing using the Dunn-Bonferroni test for multiple comparisons indicates the mean ranks were statistically significant between drug formulations A1 (Novel blend 1) and A2 (Existing blend) (p=0.007) and drug formulations A2 (Existing blend) and A3 (Novel blend 2) (p<0.001). There were no differences between drug formulations A1 (Novel blend 1) and A3 (Novel blend 2) (p=1.000).

± Post-hoc testing using the Dunn-Bonferroni test for multiple comparisons indicates the mean ranks were statistically significant between drug formulations A2 (Existing blend) and A3 (Novel blend 2) (p=0.002). There were no statistically significant differences between drug formulations A1 (Novel blend 1) and A2 (Existing blend) (p=0.076) and drug formulations A1 (Novel blend 1) and A3 (Novel blend 2) (p=0.685).

**S3: Mean Rank Comparison for Micro Labs Blends for Moxifloxacin by Age Group**

|  | **5 – 8 years old** | | | **9 – 12 years old** | | | **13 – 17 years old** | | |
| --- | --- | --- | --- | --- | --- | --- | --- | --- | --- |
|  | Rank Sum | Mean Rank  (SD) | Friedman’s F (p-value) | Rank Sum | Mean Rank  (SD) | Friedman’s F (p-value) | Rank Sum | Mean Rank  (SD) | Friedman’s F (p-value) |
|  |  |  |  |  |  |  |  |  |  |
| Overall, which Micro Labs moxifloxacin formulation did the child prefer to take? |  |  |  |  |  |  |  |  |  |
| B1 (Existing blend) | 63 | 2.03 (0.87) |  | 79 | 2.47 (0.72) |  | 82 | 2.65 (0.66) |  |
| B2 (Novel blend 2) | 62 | 2.00 (0.86) |  | 54 | 1.69 (0.74) |  | 49 | 1.58 (0.72) |  |
| B3 (Novel blend 1) | 61 | 1.96 (0.75) | 0.065 (0.968) | 59 | 1.84 (0.81) | 10.938 (0.004)** | 55 | 1.77 (0.67) | 19.936 (<0.001) # |
|  |  |  | (n=31) |  |  | (n=32) |  |  | (n=31) |
|  |  |  |  |  |  |  |  |  |  |

** Post-hoc testing using the Dunn-Bonferroni test for multiple comparisons indicates the mean ranks were statistically significant between drug formulations B1 (Existing blend) and B2 (Novel blend 2) (p<0.001) and drug formulations B1 (Existing blend) and B3 (Novel blend 1) (p=0.007). There were no differences between drug formulations B2 (Novel blend 2) and B3 (Novel blend 1) (p=1.000).

# Post-hoc testing using the Dunn-Bonferroni test for multiple comparisons indicates the mean ranks were statistically significant between drug formulations B1 (Existing blend) and B2 (Novel blend 2) (p<0.001) and drug formulations B1 (Existing blend) and B3 (Novel blend 1) (p<0.001). There were no differences between drug formulations B2 (Novel blend 2) and B3 (Novel blend 1) (p=1.000).

**S4. Mean Rank Comparison for Micro Labs Blends for Moxifloxacin by Ethnicity**

|  | **Black urban** | | | **Black rural** | | |
| --- | --- | --- | --- | --- | --- | --- |
|  | Rank Sum | Mean Rank  (SD) | Friedman’s F (p-value) | Rank Sum | Mean Rank  (SD) | Friedman’s F (p-value) |
|  |  |  |  |  |  |  |
| Overall, which Micro Labs moxifloxacin formulation did the child prefer to take? |  |  |  |  |  |  |
| B1 (Existing blend) | 71 | 2.45 (0.74) |  | 38 | 2.38 (0.72) |  |
| B2 (Novel blend 2) | 51 | 1.76 (0.79) |  | 31 | 1.94 (0.93) |  |
| B3 (Novel blend 1) | 52 | 1.79 (0.77) | 8.759 (0.013)** | 27 | 1.69 (0.70) | 3.875 (0.144) |
|  |  |  | (n=29) |  |  | (n=16) |
|  |  |  |  |  |  |  |

|  | **Coloured** | | | **Indian** | | |
| --- | --- | --- | --- | --- | --- | --- |
|  | Rank Sum | Mean Rank  (SD) | Friedman’s F (p-value) | Rank Sum | Mean Rank  (SD) | Friedman’s F (p-value) |
|  |  |  |  |  |  |  |
| Overall, which Micro Labs moxifloxacin formulation did the child prefer to take? |  |  |  |  |  |  |
| B1 (Existing blend) | 61 | 2.44 (0.87) |  | 54 | 2.25 (0.85) |  |
| B2 (Novel blend 2) | 42 | 1.68 (0.69) |  | 41 | 1.71 (0.81) |  |
| B3 (Novel blend 1) | 47 | 1.88 (0.73) | 7.760 (0.021)# | 49 | 2.04 (0.75) | 3.583 (0.167) |
|  |  |  | (n=25) |  |  | (n=24) |
|  |  |  |  |  |  |  |

** Post-hoc testing using the Dunn-Bonferroni test for multiple comparisons indicates the mean ranks were statistically significant between drug formulations B1 (Existing blend) and B2 (Novel blend 2) (p=0.004) and drug formulations B1 (Existing blend) and B3 (Novel blend 1) (p=0.007). There were no statistically significant differences between drug formulations B2 (Novel blend 2) and B3 (Novel blend 1) (p=1.000).

# Post-hoc testing using the Dunn-Bonferroni test for multiple comparisons indicates the mean ranks were statistically significant between drug formulations B1 (Existing blend) and B2 (Novel blend 2) (p=0.003) and drug formulations B1 (Existing blend) and B3 (Novel blend 1) (p=0.048). There were no statistically significant differences between drug formulations B2 (Novel blend 2) and B3 (Novel blend 1) (p=1.000).

**S5: Mean Rank Comparison for Macleods Blends for Linezolid by Age Group**

|  | **5 – 8 years old** | | | **9 – 12 years old** | | | **13 – 17 years old** | | |
| --- | --- | --- | --- | --- | --- | --- | --- | --- | --- |
|  | Rank Sum | Mean Rank  (SD) | Friedman’s F (p-value) | Rank Sum | Mean Rank  (SD) | Friedman’s F (p-value) | Rank Sum | Mean Rank  (SD) | Friedman’s F (p-value) |
|  |  |  |  |  |  |  |  |  |  |
| Overall, which Macleods linezolid formulation did the child prefer to take? |  |  |  |  |  |  |  |  |  |
| C1 (Novel blend 2) | 60 | 1.94 (0.85) |  | 66 | 2.00 (0.97) |  | 64 | 2.06 (0.77) |  |
| C2 (Novel blend 1) | 59 | 1.90 (0.70) |  | 61 | 1.85 (0.76) |  | 66 | 2.13 (0.96) |  |
| C3 (Existing blend) | 67 | 2.16 (0.90) | 1.226 (0.542) | 71 | 2.15 (0.71) | 1.515 (0.469) | 56 | 1.81 (0.70) | 1.807 (0.405) |
|  |  |  | (n=31) |  |  | (n=33) |  |  | (n=31) |
|  |  |  |  |  |  |  |  |  |  |

**S6: Mean Rank Comparison for Macleods Blends for Linezolid by Ethnicity**

|  | **Black urban** | | | **Black rural** | | |
| --- | --- | --- | --- | --- | --- | --- |
|  | Rank Sum | Mean Rank  (SD) | Friedman’s F (p-value) | Rank Sum | Mean Rank  (SD) | Friedman’s F (p-value) |
|  |  |  |  |  |  |  |
| Overall, which Macleods linezolid formulation did the child prefer to take? |  |  |  |  |  |  |
| C1 (Novel blend 2) | 60 | 2.22 (0.85) |  | 35 | 1.75 (0.91) |  |
| C2 (Novel blend 1) | 46 | 1.70 (0.78) |  | 40 | 2.00 (0.79) |  |
| C3 (Existing blend) | 56 | 2.07 (0.78) | 3.852 (0.146) | 45 | 2.25 (0.72) | 2.500 (0.287) |
|  |  |  | (n=27) |  |  | (n=20) |
|  |  |  |  |  |  |  |

|  | **Coloured** | | | **Indian** | | |
| --- | --- | --- | --- | --- | --- | --- |
|  | Rank Sum | Mean Rank  (SD) | Friedman’s F (p-value) | Rank Sum | Mean Rank  (SD) | Friedman’s F (p-value) |
|  |  |  |  |  |  |  |
| Overall, which Macleods linezolid formulation did the child prefer to take? |  |  |  |  |  |  |
| C1 (Novel blend 2) | 46 | 1.92 (0.88) |  | 49 | 2.04 (0.81) |  |
| C2 (Novel blend 1) | 49 | 2.04 (0.86) |  | 51 | 2.13 (0.80) |  |
| C3 (Existing blend) | 49 | 2.04 (0.75) | 0.250 (0.883) | 44 | 1.83 (0.87) | 1.083 (0.582) |
|  |  |  | (n=24) |  |  | (n=24) |
|  |  |  |  |  |  |  |

**S7: Mean Rank Comparison for Micro Labs Blends for Linezolid by Age Group**

|  | **5 – 8 years old** | | | **9 – 12 years old** | | | **13 – 17 years old** | | |
| --- | --- | --- | --- | --- | --- | --- | --- | --- | --- |
|  | Rank Sum | Mean Rank  (SD) | Friedman’s F (p-value) | Rank Sum | Mean Rank  (SD) | Friedman’s F (p-value) | Rank Sum | Mean Rank  (SD) | Friedman’s F (p-value) |
|  |  |  |  |  |  |  |  |  |  |
| Overall, which Micro Labs linezolid formulation did the child prefer to take? |  |  |  |  |  |  |  |  |  |
| D1 (Novel blend 1) | 60 | 1.94 (0.85) |  | 65 | 1.97 (0.73) |  | 70 | 2.26 (0.73) |  |
| D2 (Novel blend 2) | 64 | 2.06 (0.81) |  | 68 | 2.06 (0.90) |  | 55 | 1.77 (0.84) |  |
| D3 (Existing blend) | 62 | 2.00 (0.82) | 0.258 (0.879) | 65 | 1.97 (0.85) | 0.182 (0.913) | 61 | 1.97 (0.84) | 3.677 (0.159) |
|  |  |  | (n=31) |  |  | (n=33) |  |  | (n=31) |
|  |  |  |  |  |  |  |  |  |  |

**S8: Mean Rank Comparison for Micro Labs Blends for Linezolid by Ethnicity**

|  | **Black urban** | | | **Black rural** | | |
| --- | --- | --- | --- | --- | --- | --- |
|  | Rank Sum | Mean Rank  (SD) | Friedman’s F (p-value) | Rank Sum | Mean Rank  (SD) | Friedman’s F (p-value) |
|  |  |  |  |  |  |  |
| Overall, which Micro Labs linezolid formulation did the child prefer to take? |  |  |  |  |  |  |
| D1 (Novel blend 1) | 55 | 2.04 (0.81) |  | 44 | 2.10 (0.77) |  |
| D2 (Novel blend 2) | 47 | 1.74 (0.81) |  | 43 | 2.05 (0.86) |  |
| D3 (Existing blend) | 60 | 2.22 (0.80) | 3.185 (0.203) | 39 | 1.86 (0.85) | 0.667 (0.717) |
|  |  |  | (n=27) |  |  | (n=21) |
|  |  |  |  |  |  |  |

|  | **Coloured** | | | **Indian** | | |
| --- | --- | --- | --- | --- | --- | --- |
|  | Rank Sum | Mean Rank  (SD) | Friedman’s F (p-value) | Rank Sum | Mean Rank  (SD) | Friedman’s F (p-value) |
|  |  |  |  |  |  |  |
| Overall, which Micro Labs linezolid formulation did the child prefer to take? |  |  |  |  |  |  |
| D1 (Novel blend 1) | 43 | 1.87 (0.81) |  | 53 | 2.21 (0.72) |  |
| D2 (Novel blend 2) | 53 | 2.30 (0.82) |  | 44 | 1.83 (0.87) |  |
| D3 (Existing blend) | 42 | 1.83 (0.78) | 3.217 (0.200) | 47 | 1.96 (0.86) | 1.750 (0.417) |
|  |  |  | (n=23) |  |  | (n=24) |
|  |  |  |  |  |  |  |

**S9: Overall Taste Experience Score of Macleods Blends for Moxifloxacin by Age Group and by Ethnicity**

|  | A1 (Novel blend 1) | A2 (Existing blend) | A3 (Novel blend 2) | p-value |
| --- | --- | --- | --- | --- |
| **Overall taste experience score (n=97)** |  |  |  |  |
| Mean (SD) | 13.6 (5.7) | 15.6 (5.8) | 12.6 (5.7) |  |
| [IQR] | [8.0, 19.0] | [11.0, 20.0] | [8.0, 17.0] | <0.001* |
| **Overall taste experience score by Age Group** |  |  |  |  |
| 5-8 years old (n=32) |  |  |  |  |
| Mean (SD) | 13.2 (6.0) | 15.4 (5.5) | 12.9 (6.9) |  |
| [IQR] | [7.5, 19.0] | [11.5, 19.0] | [7.0, 20.5] | 0.091 |
|  |  |  |  |  |
| 9-12 years old (n=33) |  |  |  |  |
| Mean (SD) | 15.0 (5.8) | 15.5 (6.8) | 12.5 (5.3) |  |
| [IQR] | [10.0, 19.0] | [8.0, 21.0] | [8.0, 17.0] | 0.005** |
|  |  |  |  |  |
| 13-17 years old (n=32) |  |  |  |  |
| Mean (SD) | 12.8 (5.1) | 16.0 (5.1) | 12.3 (4.8) |  |
| [IQR] | [8.0, 17.0] | [12.5, 20.0] | [8.0, 16.5] | 0.003± |
| **Overall taste experience score by Ethnicity** |  |  |  |  |
| Black urban (n=29) |  |  |  |  |
| Mean (SD) | 14.0 (5.3) | 16.6 (5.8) | 12.5 (5.9) |  |
| [IQR] | [10.0, 19.0] | [13.0, 21.0] | [7.0, 17.0] | 0.013# |
|  |  |  |  |  |
| Black rural (n=18) |  |  |  |  |
| Mean (SD) | 12.8 (6.6) | 15.3 (5.4) | 12.1 (5.4) |  |
| [IQR] | [7.0, 18.0] | [13.0, 19.0] | [8.0, 14.0] | 0.025ǂ |
|  |  |  |  |  |
| Coloured (n=25) |  |  |  |  |
| Mean (SD) | 11.9 (5.0) | 13.3 (5.9) | 10.6 (4.7) |  |
| [IQR] | [8.0, 15.0] | [8.0, 18.0] | [7.0, 14.0] | 0.127 |
|  |  |  |  |  |
| Indian (n=25) |  |  |  |  |
| Mean (SD) | 15.6 (5.6) | 17.1 (5.6) | 15.0 (6.0) |  |
| [IQR] | [13.0, 19.0] | [14.0, 21.0] | [11.0, 19.0] | 0.179 |

* Post hoc comparisons using Tukey’s method for multiple comparisons indicated that overall taste experience score was significantly different between drug formulations A1 (Novel blend 1) and A2 (Existing blend) (p=0.006) and formulations A2 (Existing blend) and A3 (Novel blend 2) (p<0.001). There were no statistically significant differences between drug formulation A1 (Novel blend 1) and A3 (Novel blend 2) (p=0.209).

** Post hoc comparisons using Tukey’s method for multiple comparisons indicated that overall taste experience score was significantly different between drug formulations A2 (Existing blend) and A3 (Novel blend 2) (p=0.006) and formulations A1 (Novel blend 1) and A3 (Novel blend 2) (p=0.029). There were no statistically significant differences between drug formulation A1 (Novel blend 1) and A2 (Existing blend) (p=0.829).

± Post hoc comparisons using Tukey’s method for multiple comparisons indicated that overall taste experience score was significantly different between drug formulations A1 (Novel blend 1) and A2 (Existing blend) (p=0.015) and formulations A2 (Existing blend) and A3 (Novel blend 2) (p=0.005). There were no statistically significant differences between drug formulation A1 (Novel blend 1) and A3 (Novel blend 2) (p=0.908).

# Post hoc comparisons using Tukey’s method for multiple comparisons indicated that overall taste experience score was significantly different between drug formulations A2 (Existing blend) and A3 (Novel blend 2) (p=0.011). There were no statistically significant differences between drug formulation A1 (Novel blend 1) and A2 (Existing blend) (p=0.143) and drug formulations A1 (Novel blend 1) and A3 (Novel blend 2) (0.519).

ǂ Post hoc comparisons using Tukey’s method for multiple comparisons indicated that overall taste experience score was significantly different between drug formulations A2 (Existing blend) and A3 (Novel blend 2) (p=0.026). There were no statistically significant differences between drug formulation A1 (Novel blend 1) and A2 (Existing blend) (p=0.099) and drug formulations A1 (Novel blend 1) and A3 (Novel blend 2) (0.820).

**S10: Overall Taste Experience Score of Micro Labs Blends for Moxifloxacin by Age Group and by Ethnicity**

|  | B1 (Existing blend) | B2 (Novel blend 2) | B3 (Novel blend 1) | p-value |
| --- | --- | --- | --- | --- |
| **Overall taste experience score** |  |  |  |  |
| Mean (SD) | 17.7 (5.0) | 14.9 (5.1) | 15.8 (5.3) |  |
| [IQR] | [16.0, 21.0] | [11.0, 19.0] | [12.0, 20.0] | <0.001ǂ |
| **Overall taste experience score by Age Group** |  |  |  |  |
| 5-8 years old (n=32) |  |  |  |  |
| Mean (SD) | 15.7 (5.9) | 14.7 (5.5) | 14.3 (5.5) |  |
| [IQR] | [10.5, 21.0] | [11.5, 19.0] | [9.5, 19.0] | 0.440 |
|  |  |  |  |  |
| 9-12 years old (n=33) |  |  |  |  |
| Mean (SD) | 18.3 (4.9) | 15.4 (5.7) | 17.2 (5.5) |  |
| [IQR] | [16.0, 21.0] | [11.0, 21.0] | [12.0, 21.0] | 0.030** |
|  |  |  |  |  |
| 13-17 years old (n=32) |  |  |  |  |
| Mean (SD) | 19.1 (3.2) | 14.6 (3.9) | 15.8 (4.6) |  |
| [IQR] | [17.0, 22.0] | [12.0, 18.0] | [13.0, 18.0] | <0.001± |
| **Overall taste experience score by Ethnicity** |  |  |  |  |
| Black urban (n=29) |  |  |  |  |
| Mean (SD) | 19.9 (3.0) | 16.7 (5.0) | 16.6 (5.0) |  |
| [IQR] | [18.0, 22.0] | [12.0, 21.0] | [13.0, 21.0] | 0.005# |
|  |  |  |  |  |
| Black rural (n=18) |  |  |  |  |
| Mean (SD) | 15.9 (6.0) | 14.3 (5.0) | 15.7 (5.4) |  |
| [IQR] | [13.0, 21.0] | [11.0, 17.0] | [12.0, 19.0] | 0.109 |
|  |  |  |  |  |
| Coloured (n=25) |  |  |  |  |
| Mean (SD) | 16.8 (5.3) | 13.8 (4.7) | 13.8 (5.7) |  |
| [IQR] | [13.0, 21.0] | [10.0, 18.0] | [9.0, 19.0] | 0.070 |
|  |  |  |  |  |
| Indian (n=25) |  |  |  |  |
| Mean (SD) | 17.3 (5.0) | 14.4 (5.4) | 16.9 (5.0) |  |
| [IQR] | [16.0, 21.0] | [10.0, 19.0] | [14.0, 21.0] | 0.011ǂ |

* Post hoc comparisons using Tukey’s method for multiple comparisons indicated that overall taste experience score was significantly different between drug formulations

B1 (Existing blend) and B2 (Novel blend 2) (p<0.001) and drug formulations B1 (Existing blend) and B3 (Novel blend 1) (p=0.003). There were no statistically significant differences between drug formulation B2 (Novel blend 2) and B3 (Novel blend 1) (p=0.284).

** Post hoc comparisons using Tukey’s method for multiple comparisons indicated that overall taste experience score was significantly different between drug formulations

B1 (Existing blend) and B2 (Novel blend 2) (p=0.024). There were no statistically significant differences between drug formulation B1 (Existing blend) and B3 (Novel blend 1) (p=0.537) and drug formulations B2 (Novel blend 2) and B3 (Novel blend 1) (p=0.241).

± Post hoc comparisons using Tukey’s method for multiple comparisons indicated that overall taste experience score was significantly different between drug formulations

B1 (Existing blend) and B2 (Novel blend 2) (p<0.001) and drug formulations B1 (Existing blend) and B3 (Novel blend 1) (p=0.001). There were no statistically significant differences between drug formulation B2 (Novel blend 2) and B3 (Novel blend 1) (p=0.326).

# Post hoc comparisons using Tukey’s method for multiple comparisons indicated that overall taste experience score was significantly different between drug formulations

B1 (Existing blend) and B2 (Novel blend 2) (p=0.013) and drug formulations B1 (Existing blend) and B3 (Novel blend 1) (p=0.011). There were no statistically significant differences between drug formulation B2 (Novel blend 2) and B3 (Novel blend 1) (p=0.998).

ǂ Post hoc comparisons using Tukey’s method for multiple comparisons indicated that overall taste experience score was significantly different between drug formulations

B1 (Existing blend) and B2 (Novel blend 2) (p=0.015) and drug formulations B2 (Novel blend 2) and B3 (Novel blend 1) (p=0.044). There were no statistically significant differences between drug formulation B1 (Existing blend) and B3 (Novel blend 1) (p=0.900).

**S11: Overall Taste Experience Score of Macleods Blends for Linezolid by Age Group and by Ethnicity**

|  | C1 (Novel blend 2) | C2 (Novel blend 1) | C3 (Existing blend) | p-value |
| --- | --- | --- | --- | --- |
| **Overall taste experience score (n=95)** |  |  |  |  |
| Mean (SD) | 11.7 (4.7) | 12.6 (5.2) | 13.1 (5.4) |  |
| [IQR] | [8.0, 15.0] | [8.0, 17.0] | [8.0, 18.0] | 0.029* |
| **Overall taste experience score by Age Group** |  |  |  |  |
| 5-8 years old (n=32) |  |  |  |  |
| Mean (SD) | 11.8 (4.7) | 13.5 (5.8) | 14.3 (5.8) |  |
| [IQR] | [7.5, 16.0] | [8.5, 17.5] | [10.0, 19.5] | 0.065 |
|  |  |  |  |  |
| 9-12 years old (n=33) |  |  |  |  |
| Mean (SD) | 11.6 (5.3) | 11.5 (5.0) | 13.0 (5.2) |  |
| [IQR] | [7.0, 16.0] | [7.0, 15.0] | [8.0, 18.0] | 0.208 |
|  |  |  |  |  |
| 13-17 years old (n=30) |  |  |  |  |
| Mean (SD) | 11.6 (3.9) | 12.8 (4.7) | 12.1 (5.0) |  |
| [IQR] | [10.0, 14.0] | [9.0, 17.0] | [8.0, 16.0] | 0.399 |
| **Overall taste experience score by Ethnicity** |  |  |  |  |
| Black urban (n=27) |  |  |  |  |
| Mean (SD) | 11.4 (4.5) | 12.4 (5.3) | 13.0 (6.3) |  |
| [IQR] | [8.0, 15.0] | [8.0, 15.0] | [7.0, 18.0] | 0.458 |
|  |  |  |  |  |
| Black rural (n=20) |  |  |  |  |
| Mean (SD) | 11.5 (4.9) | 14.4 (6.3) | 15.6 (5.1) |  |
| [IQR] | [7.5, 14.0] | [9.0, 20.0] | [11.0, 19.0] | 0.016** |
|  |  |  |  |  |
| Coloured (n=24) |  |  |  |  |
| Mean (SD) | 10.8 (4.7) | 10.2 (3.8) | 11.0 (4.5) |  |
| [IQR] | [7.0, 14.0] | [7.5, 13.0] | [8.0, 13.5] | 0.490 |
|  |  |  |  |  |
| Indian (n=24) |  |  |  |  |
| Mean (SD) | 13.0 (4.6) | 13.9 (4.5) | 13.5 (4.5) |  |
| [IQR] | [9.0, 18.0] | [10.0, 17.5] | [10.5, 17.0] | 0.652 |
|  |  |  |  |  |

* Post hoc comparisons using Tukey’s method for multiple comparisons indicated that overall taste experience score was significantly different between formulation C1 and C3 (Existing blend) (p=0.024). There were no statistically significant differences between drug formulation C1 (Novel blend 2) and C2 (Novel blend 1) (p=0.203) and drug formulation C2 (Novel blend 1) and C3 (Existing blend) (p=0.620).

** Post hoc comparisons using Tukey’s method for multiple comparisons indicated that overall taste experience score was significantly different between formulation C1 (Novel blend 2) and C3 (Existing blend) (p=0.014). There were no statistically significant differences between drug formulation C1 (Novel blend 2) and C2 (Novel blend 1) (p=0.107) and drug formulation C2 (Novel blend 1) and C3 (Existing blend) (p=0.658).

**S12: Overall Taste Experience Score of Micro Labs Blends for Linezolid by Age Group and by Ethnicity**

|  | D1 (Novel blend 1) | D2 (Novel blend 2) | D3 (Existing blend) | p-value |
| --- | --- | --- | --- | --- |
| **Overall taste experience score (n=95)** |  |  |  |  |
| Mean (SD) | 13.0 (5.2) | 12.4 (5.0) | 13.0 (5.4) |  |
| [IQR] | [9.0, 17.0] | [8.0, 16.0] | [8.0, 17.0] | 0.415 |
| **Overall taste experience score by Age Group** |  |  |  |  |
| 5-8 years old (n=32) |  |  |  |  |
| Mean (SD) | 13.4 (5.7) | 12.9 (5.8) | 13.6 (6.3) |  |
| [IQR] | [9.0, 19.0] | [8.0, 18.5] | [7.5, 19.0] | 0.807 |
|  |  |  |  |  |
| 9-12 years old (n=33) |  |  |  |  |
| Mean (SD) | 12.3 (5.0) | 12.0 (4.9) | 12.1 (5.1) |  |
| [IQR] | [9.0, 16.0] | [8.0, 16.0] | [8.0, 17.0] | 0.942 |
|  |  |  |  |  |
| 13-17 years old (n=30) |  |  |  |  |
| Mean (SD) | 13.4 (5.0) | 12.2 (4.4) | 13.3 (4.5) |  |
| [IQR] | [10.0, 17.0] | [9.0, 16.0] | [10.0, 16.0] | 0.354 |
| **Overall taste experience score by Ethnicity** |  |  |  |  |
| Black urban (n=26) |  |  |  |  |
| Mean (SD) | 12.3 (5.5) | 11.2 (5.4) | 12.2 (4.9) |  |
| [IQR] | [9.0, 18.0] | [7.0, 16.0] | [8.0, 16.0] | 0.586 |
|  |  |  |  |  |
| Black rural (n=21) |  |  |  |  |
| Mean (SD) | 13.2 (4.8) | 12.7 (5.0) | 12.7 (6.9) |  |
| [IQR] | [10.0, 17.0] | [10.0, 15.0] | [6.0, 19.0] | 0.914 |
|  |  |  |  |  |
| Coloured (n=24) |  |  |  |  |
| Mean (SD) | 11.5 (5.3) | 11.5 (4.7) | 11.7 (4.3) |  |
| [IQR] | [7.0, 15.5] | [7.0, 15.0] | [8.0, 14.0] | 0.975 |
|  |  |  |  |  |
| Indian (n=24) |  |  |  |  |
| Mean (SD) | 15.1 (4.7) | 14.1 (4.7) | 15.3 (4.8) |  |
| [IQR] | [11.5, 19.0] | [10.0, 18.5] | [13.0, 20.0] | 0.531 |
|  |  |  |  |  |
